# Patterns of Weight Change One Year after Delivery Are Associated with Cardiometabolic Risk Factors at Six Years Postpartum in Mexican Women

**DOI:** 10.3390/nu12010170

**Published:** 2020-01-07

**Authors:** Diana C. Soria-Contreras, Belem Trejo-Valdivia, Alejandra Cantoral, María Luisa Pizano-Zárate, Andrea A. Baccarelli, Allan C. Just, Elena Colicino, Andrea L. Deierlein, Robert O. Wright, Emily Oken, Martha María Téllez-Rojo, Ruy López-Ridaura

**Affiliations:** 1 Center for Nutrition and Health Research, National Institute of Public Health, Avenida Universidad No. 655, Santa María Ahuacatitlán, Cuernavaca, Morelos 62100, Mexico; dianacsc@gmail.com (D.C.S.-C.); bvaldivia@insp.mx (B.T.-V.); alejandra.cantoral@insp.mx (A.C.); 2 National Council on Science and Technology, Avenida Insurgentes Sur No. 1582, San José Insurgentes, Benito Juárez, Ciudad de México 08400, Mexico; 3 Division of Community Interventions Research, National Institute of Perinatology, Montes Urales No. 800, Lomas de Virreyes, Miguel Hidalgo, Ciudad de México 11000, Mexico; pizanozarate@yahoo.com; 4 Department of Environmental Health Sciences, Mailman School of Public Health, Columbia University Medical Center, 722 West 168th Street, Suite 1105E, New York, NY 10032, USA; ab4303@cumc.columbia.edu; 5 Department of Environmental Medicine and Public Health, Icahn School of Medicine at Mount Sinai, 17 East 102 Street Floor 3, New York, NY 10029, USA; allan.just@mssm.edu (A.C.J.); elena.colicino@mssm.edu (E.C.); robert.wright@mssm.edu (R.O.W.); 6 College of Global Public Health, New York University, 715 Broadway, New York, NY 10003, USA; ald8@nyu.edu; 7 Division of Chronic Disease Research Across the Lifecourse, Department of Population Medicine, Harvard Medical School and Harvard Pilgrim Health Care Institute, Landmark Center, 401 Park Drive, Suite 401 East, Boston, MA 02215, USA; emily_oken@harvardpilgrim.org; 8 National Center for Prevention Programs and Disease Control, Benjamín Franklin No. 132, Escandón, Miguel Hidalgo, Ciudad de México 11800, Mexico; ruy.lopez@salud.gob.mx

**Keywords:** postpartum weight change, postpartum weight retention, postpartum weight gain, adiposity, cardiovascular risk, *PROGRESS* cohort

## Abstract

Pregnancy is a contributor to the obesity epidemic in women, probably through postpartum weight retention (PPWR), weight gain (PPWG), or a combination of both (PPWR + WG). The contribution of these patterns of postpartum weight change to long-term maternal health remains understudied. In a secondary analysis of 361 women from the prospective cohort *PROGRESS*, we evaluated the associations between patterns of weight change one year after delivery and cardiometabolic risk factors at six years postpartum. Using principal component analysis, we grouped cardiometabolic risk factors into: (1) body mass index (BMI), waist circumference (WC), homeostatic model assessment of insulin resistance (HOMA-IR), high-density lipoprotein cholesterol (HDL-c), triglycerides (TG), and glucose; (2) systolic (SBP) and diastolic blood pressure (DBP); and (3) low-density lipoprotein cholesterol and total cholesterol. Using path analysis, we studied direct (patterns of weight change-outcomes) and indirect associations through BMI at six years postpartum. Around 60% of women returned to their pregestational weight (reference) by one year postpartum, 6.6% experienced PPWR, 13.9% PPWG, and 19.9% PPWR + WG. Women with PPWR + WG, vs. the reference, had higher BMI and WC at six years (2.30 kg/m^2^, 95% CI [1.67, 2.93]; 3.38 cm [1.14, 5.62]). This was also observed in women with PPWR (1.80 kg/m^2^ [0.80, 2.79]; 3.15 cm [−0.35, 6.65]) and PPWG (1.22 kg/m^2^ [0.53, 1.92]; 3.32 cm [0.85, 5.78]). PPWR + WG had a direct association with HOMA-IR (0.21 units [0.04, 0.39]). The three patterns of weight change, vs. the reference, had significant indirect associations with HOMA-IR, glucose, TG, HDL-c, SBP, and DBP through BMI at six years. In conclusion, women with PPWR + WG are at high-risk for obesity and insulin resistance. Interventions targeting women during pregnancy and the first year postpartum may have implications for their long-term risk of obesity and cardiovascular disease.

## 1. Introduction

In Mexico, the prevalence of obesity among women from 20 to 49 years of age increased by almost 300% over the past three decades [1]. Pregnancy-related weight changes are possible contributors to the obesity epidemic in reproductive-aged women [2,3]. Parous, compared to nulliparous women, experience a significant increment in weight and waist circumference in the years following delivery [4]. The increased adiposity following pregnancy may put women at higher risk of obesity and metabolic dysfunction. 

According to studies conducted in high-income countries, less than 50% of women return to their pregestational weight one year after delivery [5,6,7]. Postpartum weight retention (PPWR) (i.e., weight retained from pregnancy) is a common problem with disparate consequences on women’s weight. On average, women retain from 0.8 to 2.6 kg at one year postpartum [5,8,9], but up to 27% retain ≥4.5 kg [5]. In addition, few studies have shown that some women experience postpartum weight gain (PPWG) (i.e., weight gain that originates entirely during the postpartum period), or a combination of PPWR and PPWG (PPWR + WG) [10,11,12]. 

During pregnancy, fat is gained in peripheral and central sites; however, after delivery, peripheral fat is preferentially mobilized [13,14]. This change in fat distribution favors the accumulation of central adipose tissue, from which a large part represents visceral fat [14]. Therefore, it has been suggested that weight retained after pregnancy consists of visceral fat, which may be especially harmful to women’s health. Hence, it is possible that PPWR, either alone or combined with PPWG, increases the risk of metabolic dysfunction in women. 

The literature supporting the association between patterns of postpartum weight change and cardiometabolic risk factors is scarce. Different studies have shown associations between excessive gestational weight gain (GWG) and BMI, weight change from pre-pregnancy, waist circumference (WC), and systolic blood pressure (SBP), 8–16 years after delivery [15,16]. Excessive GWG has also been associated with increased odds of overweight or obesity, and central adiposity [15,16]. Given that GWG is strongly associated with PPWR [17], it has been argued that the latter is partly responsible for some of these associations [15,16]. In addition, there is evidence suggesting that PPWR at six months and weight gain from 6 to 18 months are strongly associated with weight and BMI-adjusted WC, seven years after delivery [11]. Altogether, there is evidence to support an association between PPWR, and other patterns of postpartum weight change, with long-term adiposity and cardiometabolic outcomes, however, to the best of our knowledge, no other studies have evaluated these associations. Using data from a prospective cohort study, we aimed to evaluate the associations between four mutually exclusive patterns of weight change one year after delivery (return to pregestational weight, PPWR, PPWG, and PPWR + WG) and cardiometabolic risk factors at six years postpartum. 

## 2. Materials and Methods 

### 2.1. Study Design and Participants 

This was a secondary analysis of 948 mothers participating in the Mexico City-based prospective cohort *Programming Research in Obesity, Growth, Environment and Social Stressors* (*PROGRESS*). Between 2007 and 2011, *PROGRESS* enrolled women who were ≥18 years old, in the second trimester of a singleton pregnancy (< 22 weeks), and who received health insurance and prenatal care through the Mexican Social Security System. The eligibility criteria and a description of the cohort have been published elsewhere [18,19].

Out of the 948 participating women, 515 had information to characterize their pattern of postpartum weight change (Figure S1). After excluding women who became pregnant within the first year postpartum (*n* = 14), and one woman with an extreme weight loss during pregnancy and after delivery, the sample with information on the exposure was 500. Out of these women, 391 had information on adiposity and cardiometabolic outcomes at six years postpartum (72-month visit). Of these 391 women, 101 became pregnant again between one and six years postpartum. During pregnancy, women experience physiological changes in adiposity, insulin resistance, glucose tolerance, and lipoprotein metabolism [20], and some of these alterations remain for several months after delivery [21,22]. Considering this, we excluded women who at the six-year postpartum visit were pregnant (*n* = 9) or who had given birth within the 12 months prior to the visit (*n* = 21). The analytic sample included 361 women, who were comparable to those non-analyzed (*n* = 587) in terms of demographics, anthropometric characteristics, lifestyle behaviors, and patterns of postpartum weight change. The only exceptions were the age at enrollment (27.5 vs. 26.7 years), marital status (16.1% vs. 21.3% single), and smoking during pregnancy (69.8% vs. 61.3% never smokers). 

The Committees on Ethics, Biosafety, and Research at the Mexican National Institute of Public Health, as well as the Institutional Review Boards of the participating institutions approved the cohort procedures. At enrollment as well as at the six-year visit, women provided written informed consent after all the study procedures were explained to them.

### 2.2. Measurements 

This analysis was focused on information obtained by in-person interviews in the second and third trimester of pregnancy and at 1, 6, 12 and 72 months postpartum. A description of the measurements is presented below.

#### 2.2.1. Exposure: Patterns of Postpartum Weight Change

We categorized postpartum weight change into four mutually exclusive patterns using pregestational weight and weights measured at 1, 6, and 12 months postpartum. For this classification, the following definitions were used:

- Return to pregestational weight: Women who returned to their pregestational weight at 12 months postpartum. This group included women who lost weight compared to the pregestational state.
- Postpartum weight retention: Women who, on average, lost weight through 12 months postpartum without ever reaching their pregestational weight.
- Postpartum weight gain: Women who reached their pregestational weight at any point during the first six months postpartum and gained weight thereafter.
- Postpartum weight retention and weight gain: Women who did not return to their pregestational weight during the first six months and, on average, gained weight through 12 months postpartum.

Return to pregestational weight at any postpartum time point was defined as a weight no more than 500 g higher than pregestational weight. Pregestational weight was estimated from a prediction model as described in the Covariates Section 2.2.3. For a subset of women, we used an imputed weight at 12 months as previously reported (Soria-Contreras DC et al. Submitted for publication; 2019).

#### 2.2.2. Outcomes: Cardiometabolic Risk Factors

At six years postpartum, trained nurses measured women’s WC and weight (digital scale). WC was measured above the iliac crest to the nearest 0.1 cm with a fiberglass tape. These measurements were taken in duplicate, following standardized procedures [23]. In the case of differences ±0.2 kg for weight or ±0.5 cm for WC, they took an additional measurement. The average of the measurements was used for analysis. Height was measured at the first study visit following standardized procedures [23]. We calculated BMI at six years postpartum as weight divided by height squared. Nurses recorded SBP and diastolic blood pressure (DBP) two times, three minutes apart, with an ambulatory blood pressure monitor (Spacelabs 90217). The average was used for analysis.

Nurses collected fasting blood samples from which plasma glucose, total cholesterol (total-c) and triglycerides (TG) were assayed by enzymatic procedures using an automated analyzer (DiaSys Respons^®^910). High-density lipoprotein cholesterol (HDL-c) was assayed by an immunoprecipitation-based method (Immuno FS, DiaSys Respons^®^910). Insulin was determined by a solid-phase, enzyme-labeled chemiluminescent immunometric assay (Siemens IMMULITE 1000). We calculated insulin resistance with the homeostatic model assessment of insulin resistance (HOMA-IR) = (fasting insulin [uU/mL] × fasting glucose [mmol/L]/22.5), and low-density lipoprotein cholesterol (LDL-c) with the Friedewald equation [24].

#### 2.2.3. Covariates

At enrollment, women reported their age, parity, marital status, and education (basic: elementary and secondary school; middle: high school; and college: at least college). Using a validated questionnaire [25], women provided information on household assets, conditions (i.e., housing quality, services, and material goods), and head of household education. With this information, they were classified into six socioeconomic status (SES) categories (A/B (highest), C+, C, D+, D, and E). For this analysis, we collapsed the categories into three: high (A/B, C+, and C), middle (D+) and low (D and E). Women also provided information on smoking habits, and time spent in sedentary activities such as reading and television viewing. 

We derived mean pregnancy SBP and DBP as the average of second and third trimesters’ readings of SBP and DBP, respectively. Women were classified as being diagnosed with a hypertensive disorder of pregnancy (HDP) (preeclampsia and gestational hypertension) using information collected from their medical record. Gestational age at delivery was calculated from the child’s birth date and the self-reported last menstrual period (LMP). The Capurro method was used as a secondary method to estimate gestational age. In cases where the two methods differed by >3 weeks, the Capurro method-derived gestational age was preferred [26]. Newborn’s size for gestational age was determined by calculating the birth weight for gestational age and sex z-score, using the reference data of the International Fetal and Newborn Growth Consortium for the 21st Century [27]. A birth weight z-score >90th percentile was considered large for gestational age (LGA).

Women self-reported their pregestational weight at the first study visit (second trimester). However, given that this indicator tends to be misreported [28], we used an estimated pregestational weight obtained from a linear mixed-effects model. The model used weights measured during pregnancy (second and third trimester), as well as clinical weight measurements in the six months prior to pregnancy through the early pregnancy period that were recovered from clinical records. It also included days of gestation at the time of weight collection, maternal height, age, SES, education, parity, and self-reported pregestational weight. Model’s predictions of weight at the LMP were validated against weights objectively measured at the Mexican Social Security System clinics for a subset of women (measured within ±20 days of the LMP, *n* = 87). The predictive accuracy assessed by the root mean square error was 3.2 kg. In a post hoc analysis, we compared the model’s predictions with those obtained from a model recently proposed by Thomas et al. [28]. A Bland–Altman plot showed good agreement between the two methods (data not shown). In addition, the correlation between the two predictions was high (r = 0.99), while the average difference was low (0.16 ± 1.72 kg).

We calculated pregestational BMI as kg/m^2^ and GWG as the difference between weight measured in the third trimester of pregnancy and estimated pregestational weight. Women were classified as having adequate, insufficient, or excessive GWG for their gestational age following the United States (US) Institute of Medicine guidelines [29,30]. Using information reported via questionnaire at one month postpartum, we classified women as breastfeeding (any type) or not. 

At six years postpartum, women reported information on current smoking (yes or no), current use of medications for diabetes (metformin and/or insulin) or hypertension (yes/no), and pregnancies occurring between one and six years postpartum. Women filled-in a semi-quantitative food frequency questionnaire (FFQ) that queried the consumption of 109 food items over the past week. The FFQ was validated and used in the 2006 Mexican Health and Nutrition Survey [31]. From the FFQ, we derived total energy intake (kcal) and servings of sugar-sweetened beverages (SSBs) per day. We considered SSBs as the sum (in mL) of consumed regular soda, fruit drinks, fruit or flavored water with sugar, and coffee or tea with added sugar. Total mL per day was divided by 355 mL to represent a standard serving. We focused on SSBs because of their excessive contribution to caloric intake in Mexican adults and their consistent association with the metabolic syndrome [32,33].

### 2.3. Statistical Analysis 

All outcomes were modeled as continuous, with logarithmic transformations for glucose, HOMA-IR, HDL-c, TG, total-c, and LDL-c. Given the complex patterns of associations among the outcomes, we simplified the analysis by reducing the number of variables using a principal component approach. We identified three possible, non-correlated groups that explained 67% of the total variance. Group 1 included BMI, WC, glucose, HOMA-IR, HDL-c, and TG; Group 2 included SBP and DBP; and Group 3 included total-c and LDL-c.

We used a path analysis approach to evaluate the associations between the patterns of postpartum weight change and each group of outcome variables. One of the advantages of path analysis is that direct and indirect associations among variables can be tested [34]. In our analysis, we evaluated direct associations between the patterns of postpartum weight change and each outcome, and indirect associations mediated through BMI at six years for glucose, HOMA-IR, HDL-c, TG, SBP, DBP, total-c, and LDL-c. 

For each outcome group, we constructed a complete initial model that included covariates derived from the literature (Figure S2). In all outcome groups, we included potential baseline and pregnancy-related covariates such as sociodemographic information, pregestational BMI, GWG, breastfeeding, time in sedentary activities, and smoking during pregnancy. We also included the diagnosis of HDP because it has been associated with postpartum cardiovascular risk factors [35]. In the three groups, some post-exposure covariates were included because they may act as mediator–outcome confounders (i.e., associated with BMI and outcomes at six years) or because they may be associated with the outcomes. These included pregnancy during follow-up, smoking, energy intake, and servings of SSBs at six years postpartum. The model for Group 1 additionally included having had a newborn LGA (study pregnancy), which has been associated with postpartum adiposity, glucose, insulin, and TG [35], and use of medications for diabetes at six years. The model for Group 2 additionally included mean SBP and DBP during pregnancy and the use of medications for hypertension at six years postpartum. A reduced model for each group of outcomes was achieved by eliminating all associations with a significance level > 0.20. The robustness and fit of the reduced model were tested against the initial model, using the likelihood ratio test and Akaike’s Information Criterion. 

WC and BMI are both associated with cardiovascular mortality in the general population [36,37] and were strongly correlated (r = 0.84) in our study. We did not choose WC as a mediator for our primary analysis because of its lower precision, especially when measured above the iliac crest [38,39]. However, in a sensitivity analysis, we evaluated the indirect associations between the patterns of postpartum weight change and cardiometabolic risk factors substituting BMI at six years by WC as a mediator. The results were comparable with those presented in our main analysis and are thus not described in further detail (data not shown).

We performed all analyses using Stata 15 (StataCorp LLC, College Station, TX, USA).

## 3. Results

At baseline, women were on average 27 years, most of them were married (83.9%), low SES (54.3%), multiparous (66.5%), had basic education only (42.7%), and had never been smokers (69.8%) (Table 1). Women were on average overweight (26.5 kg/m^2^) before pregnancy, had adequate weight gain during pregnancy (46.9%), and were breastfeeding at one month (85.8%). At one year postpartum, 59.6% had returned to their pregestational weight, while 6.6% experienced PPWR, 13.9% PPWG, and 19.9% PPWR + WG. Marital status, parity, and GWG differed by the pattern of postpartum weight change (*p* < 0.05). 

Figure S3 displays the average BMI (unadjusted) by pattern of postpartum weight change from 12 to 72 months. On average, women showed a trend to increase BMI from 12 to 72 months, with some variability by pattern of postpartum weight change. Women who returned to their pregestational weight by one year postpartum showed the greatest increase in BMI from 12 to 72 months (1.9 kg/m^2^), while those with PPWG and PPWR + WG experienced the lowest increase (~1 kg/m^2^). Despite this, women who returned to their pregestational weight had the lowest increase in BMI from pre-pregnancy to 72 months with a mean of 0.6 kg/m^2^; those with PPWR + WG had the highest increase from pre-pregnancy with a mean of 3.3 kg/m^2^.

**Figure 1:**
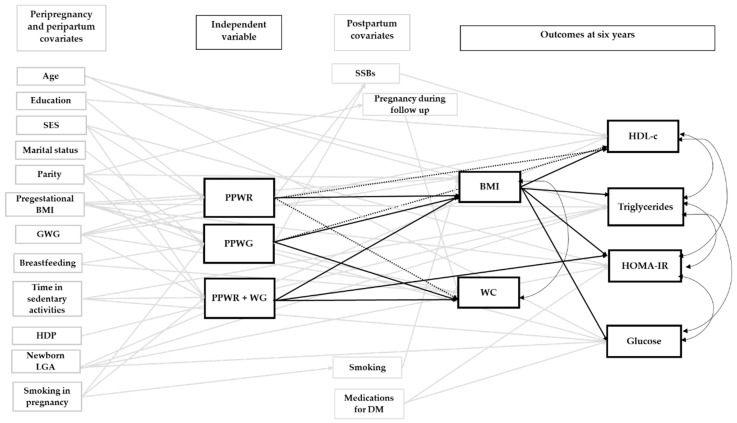
Final path model of the association between patterns of weight change one year after delivery and Outcome Group 1 at six years postpartum. The figure displays direct associations between patterns of postpartum weight change and Outcome Group 1, and indirect associations mediated through BMI. The reference group was women who returned to their pregestational weight by one year postpartum. Arrows represent pathways among variables. The black arrows represent paths relevant to our study objectives. A continuous black arrow indicates a statistically significant association (*p* < 0.05); a doted black arrow represents a tendency of association (0.05 ≤ *p* < 0.10). BMI, body mass index; DM, diabetes mellitus; GWG, gestational weight gain; HDL-c, high-density lipoprotein cholesterol; HDP, hypertensive disorders of pregnancy; HOMA-IR, homeostatic model assessment of insulin resistance; LGA, large for gestational age; PPWG, postpartum weight gain; PPWR, postpartum weight retention; PPWR + WG, postpartum weight retention + weight gain; SES, socioeconomic status; SSBs, sugar-sweetened beverages; WC, waist circumference.

**Figure 2:**
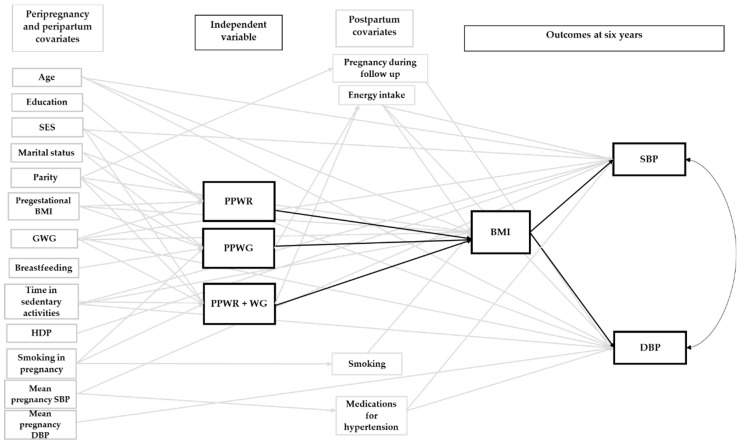
Final path model of the association between patterns of weight change one year after delivery and Outcome Group 2 at six years postpartum. The reference group was women who returned to their pregestational weight by one year postpartum. Arrows represent pathways among variables. The black arrows represent paths relevant to our study objectives. A continuous black arrow indicates a statistically significant association (*p* < 0.05). For these outcomes, only indirect associations (mediated through BMI) were identified. BMI, body mass index; DBP, diastolic blood pressure; GWG, gestational weight gain; HDP, hypertensive disorders of pregnancy; PPWG, postpartum weight gain; PPWR, postpartum weight retention; PPWR + WG, postpartum weight retention + weight gain; SBP, systolic blood pressure; SES, socioeconomic status.

**Figure 3:**
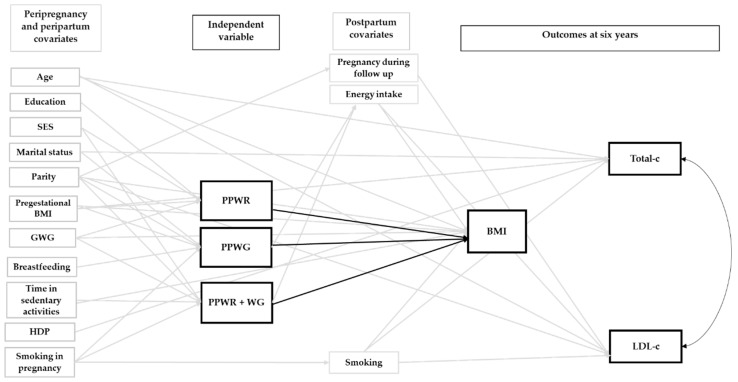
Final path model of the association between patterns of weight change one year after delivery and Outcome Group 3 at six years postpartum. The reference group was women who returned to their pregestational weight by one year postpartum. Arrows represent pathways among variables. The black arrows represent paths relevant to our study objectives. A continuous black arrow indicates a statistically significant association (*p* < 0.05). For these outcomes, neither direct nor indirect associations were identified. BMI, body mass index; GWG, gestational weight gain; HDP, hypertensive disorders of pregnancy; LDL-c, low-density lipoprotein cholesterol; PPWG, postpartum weight gain; PPWR, postpartum weight retention; PPWR + WG, postpartum weight retention + weight gain; SES, socioeconomic status; total-c, total cholesterol.

Figure S2 displays the initial model for all the outcome groups. As mentioned above, this model included relevant pre-pregnancy, gestational and postpartum covariates identified after a thorough literature review. Figure 1, Figure 2 and Figure 3 show the final adjusted path models for Outcome Groups 1–3, respectively. The gray arrows represent paths (*p* < 0.20) between the covariates, and the patterns of weight change or cardiometabolic outcomes, which were not of primary interest. The black arrows (continuous and doted, *p* < 0.05 and 0.05 ≤ *p* < 0.10, respectively) represent the paths central to our research objective (i.e., patterns of postpartum weight change and outcomes at six years). For BMI and WC, we only tested direct associations with the patterns of postpartum weight change, whereas, for glucose, TG, HOMA-IR, HDL-c, SBP, DBP, total-c, and LDL-c, direct and indirect associations (through BMI) were tested. Taking the association of PPWR and HDL-c as an example (Figure 1), it can be observed that there is a doted black arrow between these variables, which shows a tendency (0.05 ≤ *p* < 0.10) of a direct association. On the other hand, PPWR and HDL-c are indirectly associated, through BMI, at six years as shown by the continuous black arrow (*p* < 0.05) between PPWR and BMI, and BMI and HDL-c at six years.

Figure 1 displays the direct and indirect associations between the patterns of postpartum weight change and Outcome Group 1. The corresponding direct and indirect *β* coefficients are presented in Table 2. PPWR, PPWG, and PPWR + WG were directly associated with BMI and WC at six years postpartum. Compared to the reference group, women who experienced PPWR + WG had the greatest BMI at six years (*β* 2.30 kg/m^2^, 95% CI [1.67, 2.93]), followed by women with PPWR (1.80 kg/m^2^ [0.80, 2.79]) and PPWG (1.22 kg/m^2^ [0.53, 1.92]). For WC, the increase was similar for the three groups with 3.38 cm [1.14, 5.62], 3.32 cm [0.85, 5.78], and 3.15 cm [−0.35, 6.65] for PPWR + WG, PPWG, and PPWR, respectively. However, for PPWR, the 95% CI for WC included the null value. 

PPWR + WG had a significant, direct association with HOMA-IR, while for PPWR and PPWG the direct pathways were not significant (Figure 1). Compared to women who returned to their pregestational weight by one year postpartum, those with PPWR + WG had a higher HOMA-IR (0.21 units [0.04, 0.39]), which translates to 23% higher levels (Table 2). The indirect pathways, mediated by BMI at six years, were significant for all the patterns of postpartum weight change (Figure 1) and corresponded to 20%, 15%, and 11% higher insulin resistance for PPWR + WG, PPWR, and PPWG, respectively (Table 2). PPWR and PPWG showed tendencies of direct associations with HDL-c (Figure 1). As shown in Table 2, these associations corresponded to 10% and 7% lower HDL-c for PPWR and PPWG, respectively. The three patterns of postpartum weight change had significant associations with HDL-c mediated through BMI (Figure 1). These associations were similar in magnitude and corresponded to a 3% lower HDL-c for PPWR + WG and PPWR, and 2% lower for PPWG, compared to the reference group (Table 2). As Figure 1 shows, we did not find direct associations between any of the patterns of weight change with glucose and TG, but indirect associations, through BMI, were identified. Compared to the reference group, women with PPWR + WG had higher concentrations of glucose (3%) and TG (15%). The same was observed for women with PPWR (+ 2% glucose, +12% TG) and PPWG (+ 1% glucose, +8% TG) (Table 2). 

Figure 2 and Figure 3 show the direct and indirect associations between the patterns of postpartum weight change and Outcome Groups 2 and 3. The corresponding direct and indirect *β* coefficients are presented in Table 3. For SBP and DBP, only the indirect pathways, mediated through BMI, were significant (Figure 2). Women with PPWR + WG had the highest SBP (1.11 mm Hg [0.53, 1.70]) and DBP (0.61 mm Hg [0.16, 1.06]), followed by women with PPWR (SBP, 0.90 mm Hg [0.26, 1.54]; DBP, 0.50 mm Hg [0.06, 0.93]) and PPWG (SBP, 0.62 mm Hg [0.18, 1.07]; DBP, 0.34 mm Hg [0.04, 0.65]) (Table 3). For LDL-c and total-c, we did not find significant direct or indirect pathways (Figure 3 and Table 3). 

## 4. Discussion

This study provides the first evidence on the effect of postpartum weight change patterns in relation to long-term adiposity and cardiovascular risk. In this cohort of Mexican women, those who experienced PPWR + WG, PPWR, and PPWG, compared to women who returned to their pregestational weight, had increased adiposity six years after delivery. These three patterns of postpartum weight change were indirectly associated, through BMI, with cardiovascular risk factors at six years. Only PPWR + WG had a significant, direct association with insulin resistance six years after delivery, suggesting that women who followed this pattern of weight change may be particularly at increased cardiovascular risk. 

PPWR + WG, PPWR, and PPWG one year after delivery, compared to the reference group, were associated with increased BMI and WC at six years postpartum, with stronger associations for PPWR + WG and PPWR, especially for BMI. The association between PPWR and WC was strong but not highly significant probably because of the small sample in this group (*n* = 24). The findings for PPWR + WG and PPWR are supported by previous research that showed associations between excessive GWG and long-term adiposity outcomes [15,16,40,41]. McClure et al., in a study of American women showed that excessive GWG, compared to adequate, was associated with a 4.9% higher BMI, and a 3.2 cm higher WC at an average of eight years postpartum [16]. Although our results are not directly comparable, we could argue that the pattern of postpartum weight change, in specific PPWR + WG or PPWR, might be the potential link between excessive GWG and long-term adiposity. In fact, we previously showed an increased risk of PPWR + WG and PPWR one year after delivery in women with excessive GWG (Soria-Contreras DC et al. Submitted for publication; 2019). This mechanistic explanation is further supported by findings from 23,701 women within the Danish National Birth Cohort, which showed that GWG was associated with maternal weight at seven years postpartum primarily through PPWR. In this study, 1 kg of weight retained at six months corresponded to a 0.48-kg increase in weight and to a 0.03-cm higher BMI-adjusted WC at seven years postpartum, independently of GWG [11]. In our previous analysis, pregestational overweight and obesity increased the risk of PPWG (Soria-Contreras DC et al. Submitted for publication; 2019). Behavioral factors associated with long-term weight gain such as lack of physical activity, longer time in sedentary activities, and poor diet quality are more prevalent among women with overweight or obesity [42,43,44]. Therefore, the association between PPWG and long-term adiposity may be the result of pre-existing unhealthy behaviors and weight-control challenges in these women. 

In our study, we did not find significant direct associations between any of the patterns of weight change with glucose, TG, SBP, DBP, total-c, and LDL-c. PPWR + WG had a direct and significant association with HOMA-IR at six years postpartum. Previous studies have failed to show associations between HOMA-IR and total, trimester-specific [41], or excessive GWG [16]. One possible explanation for these inconsistencies is that GWG reflects increases in different components not particularly related to metabolic risk, including placental and fetal weight, as well as fat-free mass and fat mass [30]. On the other hand, PPWR + WG might reflect primarily increases in fat mass, including visceral adipose tissue, which is associated with insulin resistance [45]. It is not surprising that only women with PPWR + WG had higher insulin resistance since they experienced the highest weight (7.7 kg) and BMI (3.3 kg/m^2^) increase from pre-pregnancy to six years postpartum. According to one study of non-pregnant French women and men, weight gain over six years was associated with worsening in all metabolic syndrome components especially WC and insulin. Women who gained 6–8 kg increased their WC by 8% and their insulin concentrations by ~30%, compared to those who maintained their weight stable (± 2 kg) [46]. 

PPWR and PPWG had marginally significant direct associations with low HDL-c (0.05 ≤ *p* < 0.10). For PPWR + WG the association was in the same direction but the 95% CI was wider (0.10 ≤ *p* < 0.20). In a recent study among Latina women in the US, each additional live birth was associated with increased odds of both abdominal obesity and low HDL-c (OR 1.1, 95% CI 1.0–1.2) [47]. It has been postulated that an increase in adiposity, specifically visceral adipose tissue, following pregnancy may in part be responsible for the associations between childbearing and low HDL-c [47,48,49]; however, this has not been confirmed. This would explain why in our study women in any of the three patterns of postpartum weight change had similar WC at six years postpartum, as well as lower HDL-c. However, the fact that PPWR + WG had the strongest association with BMI and WC, and the weakest association with HDL-c deserves further investigation.

PPWR + WG, PPWR, and PPWG had significant indirect associations with glucose, TG, HOMA-IR, HDL-c, SBP, and DBP through BMI at six years postpartum. These are expected findings given that a higher BMI is associated with higher levels of all the components of the metabolic syndrome, and lower concentrations of HDL-c [50,51]. Overall, the associations were stronger for PPWR + WG, followed by PPWR and PPWG, which reflects the stronger association of PPWR + WG with BMI at six years. On the other hand, we determined LDL-c with the Friedewald formula instead of direct quantification. The underestimation of LDL-c levels may explain the lack of indirect association with this risk factor [52].

This study adds to the literature on the effect of pregnancy on long-term health outcomes in several ways. First, we showed that, independently of GWG, PPWR + WG, PPWR, and PPWG are associated with long-term adiposity, which may put women at higher risk of cardiovascular disease. Second, the most prevalent pattern of postpartum weight change was a combination of PPWR + WG. This pattern was also associated with the worst cardiometabolic profile at six years postpartum, characterized by increased BMI, WC, and insulin resistance. Increased adiposity and insulin resistance are central components of the metabolic syndrome; therefore, women with PPWR + WG may be a higher risk group for developing this syndrome. 

To date, different prenatal weight management interventions have been successful in reducing GWG but not weight one year after delivery [53,54,55]. One reason for this may be that, as shown in this study, postpartum weight not only consists of weight retained from pregnancy but also weight gain that originates during the postpartum period. For instance, limiting GWG may be effective for reducing PPWR but may not be sufficient to prevent PPWG and, to some extent, PPWR + WG. Therefore, extending weight management interventions through the first year after delivery may have the potential to benefit women at risk of weight gain. This is supported by the results of a recent analysis of the LIFE-Moms lifestyle intervention clinical trials. In this study, prenatal interventions continued in the postpartum period were the most successful in reducing weight by one year postpartum (mean difference of −1.9 kg vs. −1.1 kg of prenatal intervention only). Behavioral lifestyle interventions focused on healthy eating, physical activity, and behavioral weight management strategies were the most effective in reducing postpartum weight [56]. As most of the studies have been conducted among women in the US, future research is warranted to define the optimal intervention among Latin American women outside the US.

This work has some limitations and strengths that must be considered. Our population was primarily low SES women living in Mexico City, which limits the generalizability of our findings. In this analysis, we used information collected throughout more than six years of follow-up. During this time, some women were lost to follow-up or had some missing information; therefore, only 38% of women participating in *PROGRESS* had enough information to be included in the present analysis. However, included women were not substantially different from those excluded. As in most studies, confounding cannot be completely ruled out. We minimized this possibility by adjusting our path models for relevant covariates, but some important information was missing. For example, we did not have information on the maternal glucose tolerance status or the diagnosis of gestational diabetes during the *PROGRESS* pregnancy, or in any subsequent pregnancy. Instead, we adjusted by the newborn’s size for gestational age at the study pregnancy because of the strong, positive correlation between maternal glucose concentrations during pregnancy and infant birth weight [57]. We had information on medication intake at six years but not on the diagnosis of diabetes or any chronic disease. Therefore, we cannot rule out the possibility of residual confounding on these variables potentially associated with maternal outcomes at six years. Another limitation is that measured pregestational weight was not available in *PROGRESS*. Instead of using a self-reported pregestational weight that was available for all women, we used an estimated pregestational weight from a prediction model. Self-reported pregestational weight tends to be misreported but, even with some degree of error, is accurate for some populations [28]. However, this is not the case for women participating in *PROGRESS* who, according to a recent study, may be less likely to know their pregestational weight and have high ranges of misreport (−39.2 to 25.7 kg) [28]. Given the absence of measured pregestational weight, we would argue that using the predicted weight, instead of self-reported, is less subject to error in our population.

Our study has some strengths worth mentioning. Our cohort had extensive information on potential confounders and the majority of the anthropometric measures were objectively measured by trained personnel. Additionally, we used a statistical approach that allowed us to examine the direct effect of the patterns of postpartum weight change on outcomes, but also an indirect effect mediated through adiposity at six years postpartum. With this approach, we were also able to consider the temporal and complex relations between the study variables. 

## 5. Conclusions

Our results suggest that women who do not recover their pregestational weight during the first year postpartum have increased adiposity years after delivery and are at increased cardiovascular risk. Women who retain weight from pregnancy and, additionally, gain weight after delivery are, in particular, a high-risk group for obesity, insulin resistance, and the metabolic syndrome. Lifestyle interventions focused on behavioral strategies, healthy eating, and physical activity during pregnancy and the first year after delivery may have important implications for women’s long-term risk of obesity and cardiovascular disease. 

## Figures and Tables

**Table 1:** Participant characteristics according to patterns of postpartum weight change in women participating in the *PROGRESS* cohort.

	*PATTERNS OF POSTPARTUM WEIGHT CHANGE*					
	All Women *n* = 361	Return to Pregestational Weight *n* = 215 (59.6%)	PPWR *n* = 24 (6.6%)	PPWG *n* = 50 (13.9%)	PPWR + WG *n* = 72 (19.9%)	*p* ^1^
***Pre-pregnancy Characteristics***						
**Maternal Age, Years (mean ± SD)**	27.5 ± 5.6	27.6 ± 5.6	26.9 ± 4.3	27.6 ± 6.4	27.4 ± 5.4	0.94
**Pregestational BMI, kg/m^2^ (mean ± SD)**	26.5 ± 4.2	26.3 ± 4.2	25 ± 3.4	27.7 ± 4.2	26.5 ± 4.3	0.08
***n* (%)**						
**Education**						0.10
***Basic***	154 (42.7)	87 (40.5)	17 (70.8)	21 (42.0)	29 (40.3)	
***Middle***	126 (34.9)	77 (35.8)	6 (25.0)	18 (36.0)	25 (34.7)	
***College***	81 (22.4)	51 (23.7)	1 (4.2)	11 (22.0)	18 (25.0)	
**Marital status**						0.02
***Single***	58 (16.1)	26 (12.1)	2 (8.3)	14 (28.0)	16 (22.2)	
***Married***	303 (83.9)	189 (87.9)	22 (91.7)	36 (72.0)	56 (77.8)	
**SES**						0.13
***High***	86 (23.8)	59 (27.4)	4 (16.7)	14 (28.0)	9 (12.5)	
***Medium***	79 (21.9)	44 (20.5)	6 (25.0)	13 (26.0)	16 (22.2)	
***Low***	196 (54.3)	112 (52.1)	14 (58.3)	23 (46.0)	47 (65.3)	
**Parity**						0.01
***Primiparous***	121 (33.5)	60 (27.9)	6 (25.0)	21 (42.0)	34 (47.2)	
***Multiparous***	240 (66.5)	155 (72.1)	18 (75.0)	29 (58.0)	38 (52.8)	
***Pregnancy characteristics***						
**Smoking**						0.38
***Never Smokers***	252 (69.8)	152 (70.7)	15 (62.5)	36 (72.0)	49 (68.1)	
***Smokers around Pregnancy***	78 (21.6)	44 (20.5)	7 (29.2)	13 (26.0)	14 (19.4)	
***Former Smokers***	31 (8.6)	19 (8.8)	2 (8.3)	1 (2.0)	9 (12.5)	
**Sedentary activities ^2^**						0.50
**< *2 hours/day***	161 (44.6)	102 (47.4)	11 (45.8)	21 (42.0)	27 (37.5)	
**≥ *2 hours/day***	200 (55.4)	113 (52.6)	13 (54.2)	29 (58.0)	45 (62.5)	
**Adequacy of GWG**						<0.001
***Insufficient***	85 (26.2)	61 (32.1)	0 (0.0)	17 (35.4)	7 (10.9)	
***Adequate***	152 (46.9)	90 (47.4)	10 (45.5)	22 (45.8)	30 (46.9)	
***Excessive***	87 (26.9)	39 (20.5)	12 (54.5)	9 (18.8)	27 (42.2)	
**Newborn LGA**	29 (8.0)	19 (8.8)	1 (4.2)	6 (12.0)	3 (4.2)	0.33
**Diagnosis of HDP**	24 (6.7)	15 (7.0)	1 (4.2)	5 (10.0)	3 (4.2)	0.59
***Postpartum Characteristics***						
**Any Breastfeeding at 1 Month**						0.32
***Yes***	302 (85.8)	178 (86.4)	20 (83.3)	46 (92.0)	58 (80.6)	
***No***	50 (14.2)	28 (13.6)	4 (16.7)	4 (8.0)	14 (19.4)	

^1^*p*-value from multinomial logistic regression. ^2^ Includes time reading and watching television. GWG, gestational weight gain; HDP, hypertensive disorders of pregnancy (preeclampsia and gestational hypertension); LGA, large for gestational age; PPWG, postpartum weight gain; PPWR, postpartum weight retention; PPWR + WG, postpartum weight retention + weight gain; SES, socioeconomic status.

**Table 2:** Path coefficients and 95% CI for the association between patterns of weight change one year after delivery with adiposity and selected cardiometabolic outcomes at six years postpartum.

	*Direct Associations*			*Indirect Associations*		
Outcome Group 1	*β*	95% CI	*p*	*β*	95% CI	*p*
**Body Mass Index (kg/m^2^) ^1^**						
**PPWR**	1.80	0.80, 2.79	<0.001			
**PPWG**	1.22	0.53, 1.92	0.001			
**PPWR + WG**	2.30	1.67, 2.93	<0.001			
**Waist Circumference (cm) ^1^**						
**PPWR**	3.15	−0.35, 6.65	0.08			
**PPWG**	3.32	0.85, 5.78	0.008			
**PPWR + WG**	3.38	1.14, 5.62	0.003			
**Log glucose**						
**PPWR**	−0.03	−0.09, 0.02	0.27	0.02	0.004, 0.04	0.012
**PPWG**	0.002	−0.04, 0.04	0.94	0.01	0.003, 0.02	0.013
**PPWR + WG**	−0.03	−0.06, 0.01	0.16	0.03	0.01, 0.04	0.002
**Log triglycerides**						
**PPWR**	0.05	−0.17, 0.26	0.68	0.11	0.04, 0.18	0.003
**PPWG**	−0.08	−0.23, 0.06	0.26	0.08	0.03, 0.13	0.003
**PPWR + WG**	−0.07	−0.21, 0.07	0.33	0.14	0.08, 0.20	<0.001
**Log HOMA-IR**						
**PPWR**	−0.18	−0.45, 0.08	0.17	0.14	0.05, 0.24	0.003
**PPWG**	0.15	−0.03, 0.33	0.10	0.10	0.03, 0.16	0.003
**PPWR + WG**	0.21	0.04, 0.39	0.018	0.18	0.10, 0.26	<0.001
**Log HDL-c**						
**PPWR**	−0.10	−0.20, 0.01	0.08	−0.03	−0.04, −0.01	0.004
**PPWG**	−0.07	−0.15, 0.003	0.06	−0.02	−0.03, −0.01	0.004
**PPWR + WG**	−0.05	−0.12, 0.02	0.19	−0.03	−0.05, −0.02	<0.001

Reference group: return to pregestational weight. **^1^** For BMI and WC at six years only direct associations were tested. HDL-c, high-density lipoprotein cholesterol; HOMA-IR, homeostatic model assessment of insulin resistance; PPWG, postpartum weight gain; PPWR, postpartum weight retention; PPWR + WG, postpartum weight retention + weight gain.

**Table 3:** Path coefficients and 95% CI for the association between patterns of weight change one year after delivery with SBP, DBP, total-c, and LDL-c at six years postpartum.

	Direct Associations			Indirect Associations		
Outcome	*β*	95% CI	*p*	*β*	95% CI	*p*
**Group 2**						
**SBP (mm Hg)**						
**PPWR**	1.22	−2.86, 5.31	0.56	0.9	0.26, 1.54	0.006
**PPWG**	0.44	−2.47, 3.36	0.77	0.62	0.18, 1.07	0.006
**PPWR + WG**	1.09	−1.62, 3.80	0.43	1.11	0.53, 1.70	<0.001
**DBP (mm Hg)**						
**PPWR**	1.65	−1.83, 5.13	0.35	0.5	0.06, 0.93	0.025
**PPWG**	−0.90	−3.42, 1.61	0.48	0.34	0.04, 0.65	0.027
**PPWR + WG**	0.71	−1.57, 3.00	0.54	0.61	0.16, 1.06	0.008
**Group 3**						
**Log total-c**						
**PPWR**	−0.02	−0.09, 0.06	0.65	0.01	−0.003, 0.02	0.14
**PPWG**	−0.02	−0.07, 0.04	0.57	0.01	−0.002, 0.01	0.14
**PPWR + WG**	−0.01	−0.06, 0.05	0.84	0.01	−0.003, 0.03	0.11
**Log LDL-c**						
**PPWR**	−0.003	−0.13, 0.13	0.97	−0.01	−0.02, 0.01	0.4
**PPWG**	−0.05	−0.14, 0.04	0.29	−0.004	−0.01, 0.005	0.4
**PPWR + WG**	0.03	−0.06, 0.11	0.51	−0.01	−0.02, 0.01	0.39

Reference group: return to pregestational weight. DBP, diastolic blood pressure; LDL-c, low-density lipoprotein cholesterol; PPWG, postpartum weight gain; PPWR, postpartum weight retention; PPWR + WG, postpartum weight retention + weight gain; SBP, systolic blood pressure; Total-c, total cholesterol.

## Supplementary Materials

The following are available online at https://www.mdpi.com/2072-6643/12/1/170/s1, Figure S1: Flowchart of the study population, Figure S2: Initial path models of the association between patterns of weight change one year after delivery and cardiometabolic risk factors at six years postpartum, Figure S3: BMI by patterns of weight change from 12 to 72 months postpartum.
