# Supplementary material for: Patterns of Weight Change One Year after Delivery Are Associated with Cardiometabolic Risk Factors at Six Years Postpartum in Mexican Women
Source: Nutrients. 2020 Jan 7;12(1):170. doi: 10.3390/nu12010170 (PMC7019329; doi:10.3390/nu12010170)
Supplement: Supplementary file 1 [file nutrients-12-00170-s001.pdf]

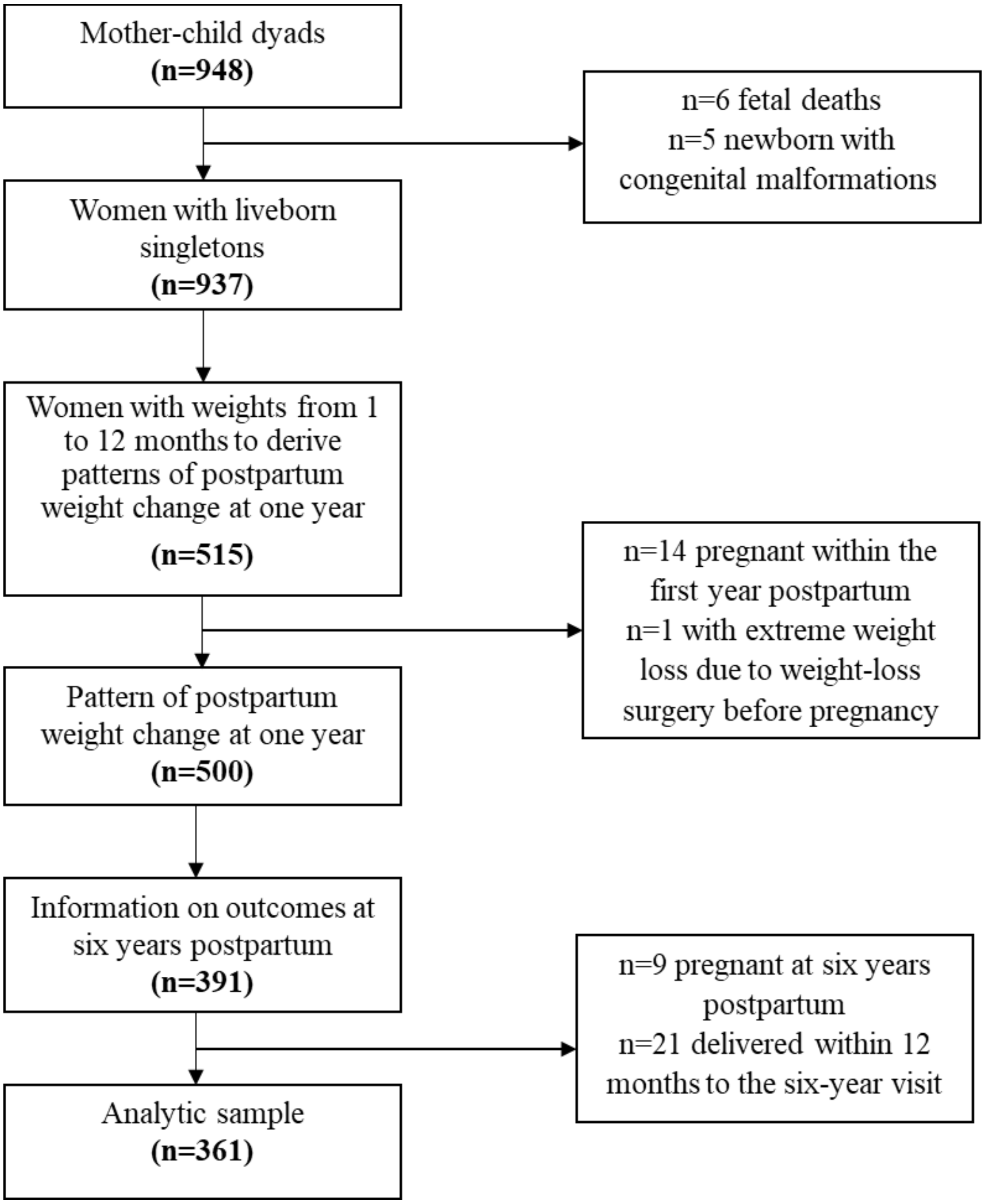

Figure S1. Flowchart of the study population

A

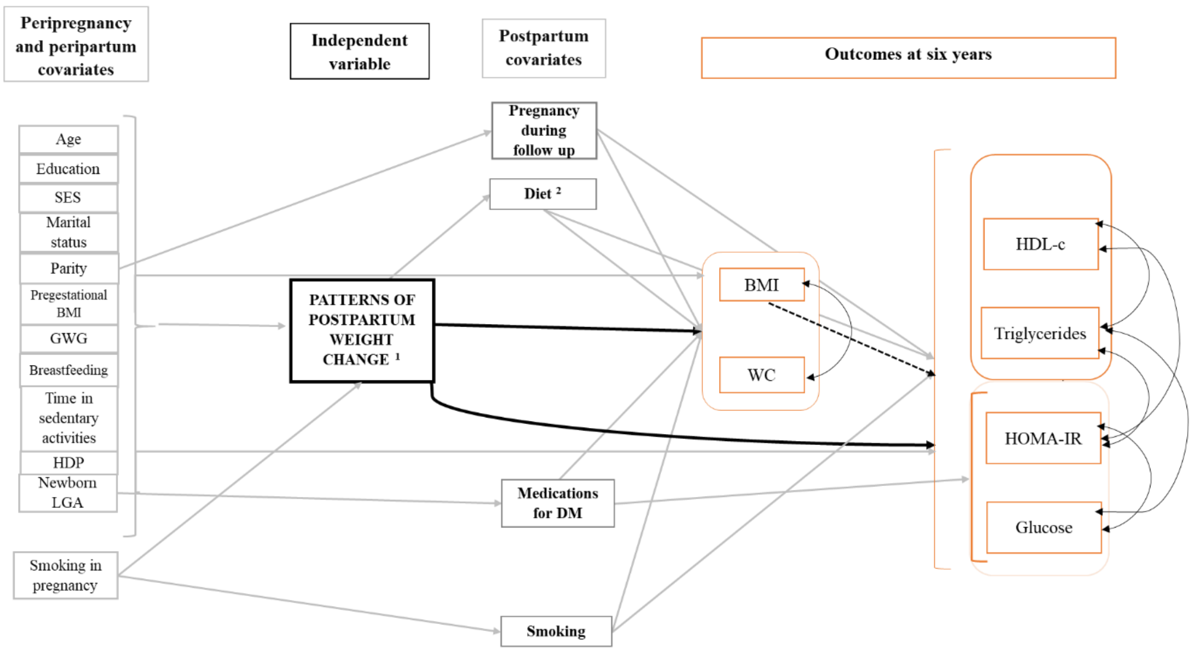
Peripregnancy and peripartum covariates
Independent variable
Postpartum covariates
Outcomes at six years
Age
Education
SES
Marital status
Parity
Pregestational BMI
GWG
Breastfeeding
Time in sedentary activities
HDP
Newborn LGA
Smoking in pregnancy
Pregnancy during follow up
Diet ²
PATTERNS OF POSTPARTUM WEIGHT CHANGE ¹
Medications for DM
Smoking
BMI
WC
HDL-c
Triglycerides
HOMA-IR
Glucose

B

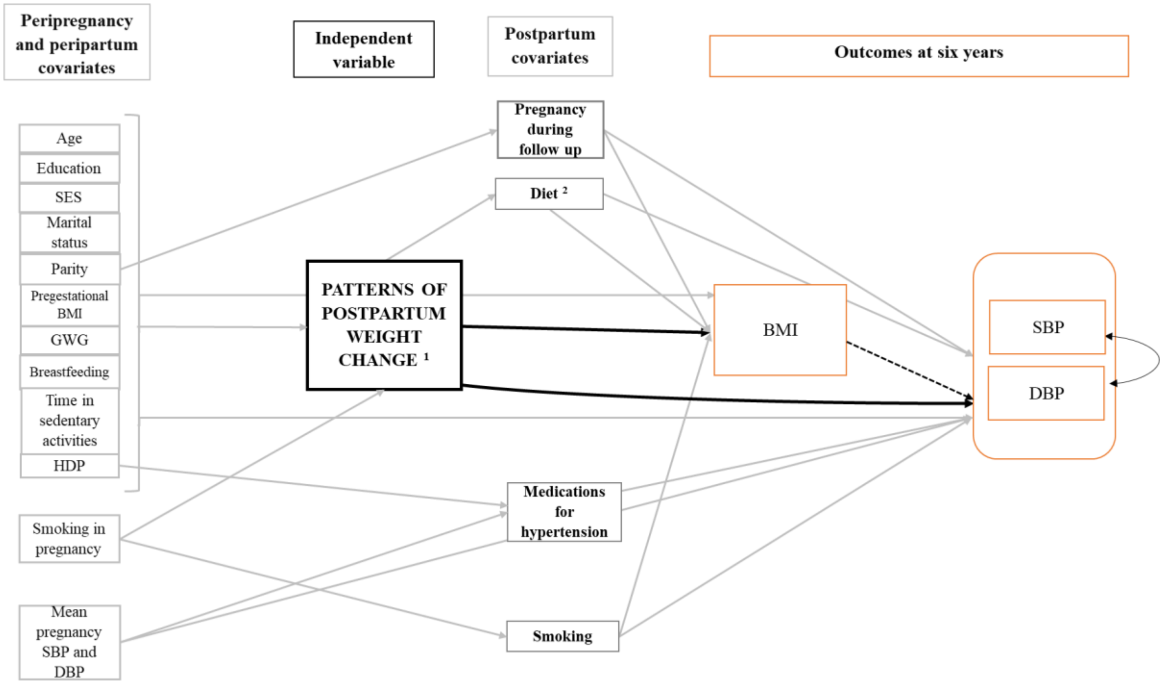
Peripregnancy and peripartum covariates
Independent variable
Postpartum covariates
Outcomes at six years
Age
Education
SES
Marital status
Parity
Pregestational BMI
GWG
Breastfeeding
Time in sedentary activities
HDP
Smoking in pregnancy
Mean pregnancy SBP and DBP
Pregnancy during follow up
Diet ²
PATTERNS OF POSTPARTUM WEIGHT CHANGE ¹
Medications for hypertension
Smoking
BMI
SBP
DBP

C

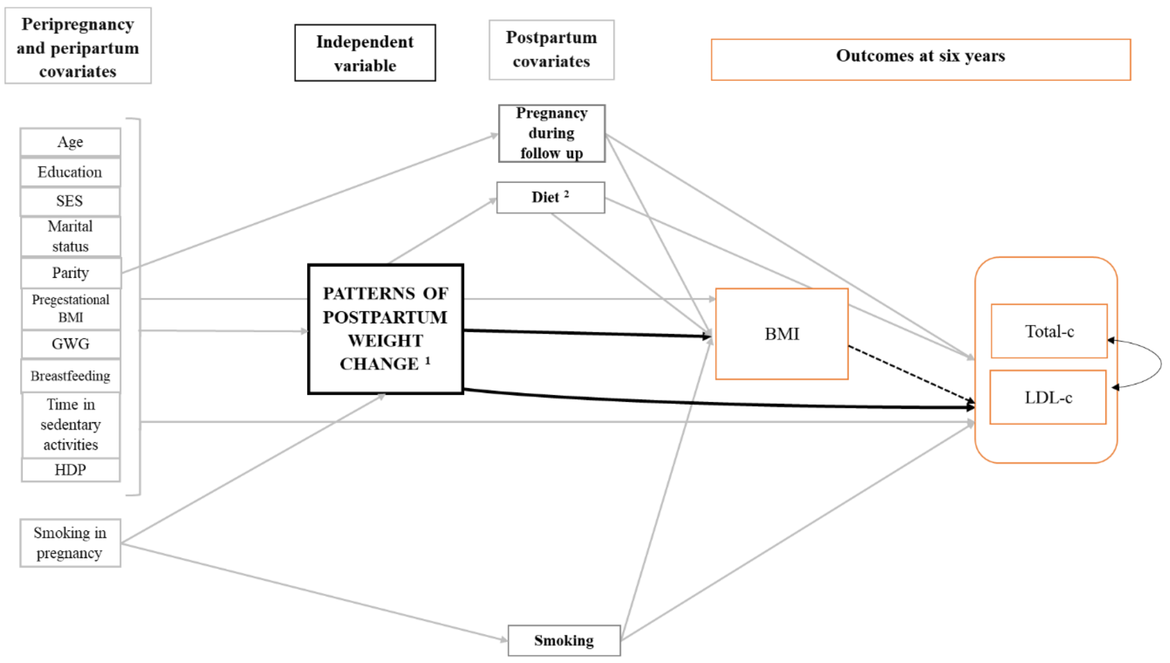

Figure S2. Initial path models of the association between patterns of weight change one year after delivery and cardiometabolic risk factors at six years postpartum.

Figure A highlights the possible paths between the patterns of postpartum weight change and outcome group 1; **figure B** focuses on outcome group 2; **figure C** refers to outcome group 3. Arrows represent pathways among variables. The black arrows represent paths relevant to our study objectives. A continuous black arrow indicates a possible direct association between the patterns of postpartum weight change and outcomes; a doted black arrow highlights a possible indirect association between patterns of weight change and outcomes, mediated through BMI at six years.

[1] Patterns of postpartum weight change included: return to pregestational weight (reference group), postpartum weight retention, postpartum weight gain, postpartum weight retention + weight gain.

[2] Diet includes energy intake per day (kcal), and servings of sugar-sweetened beverages per day at six years.

BMI, body mass index; DBP, diastolic blood pressure; DM, diabetes mellitus; GWG, gestational weight gain; HDL-c, high-density lipoprotein cholesterol; HDP, hypertensive disorders of pregnancy; HOMA-IR, homeostatic model assessment of insulin resistance; LDL-c, low-density lipoprotein cholesterol; LGA, large for gestational age; SBP, systolic blood pressure; SES, socioeconomic status; total-c, total cholesterol; WC, waist circumference.

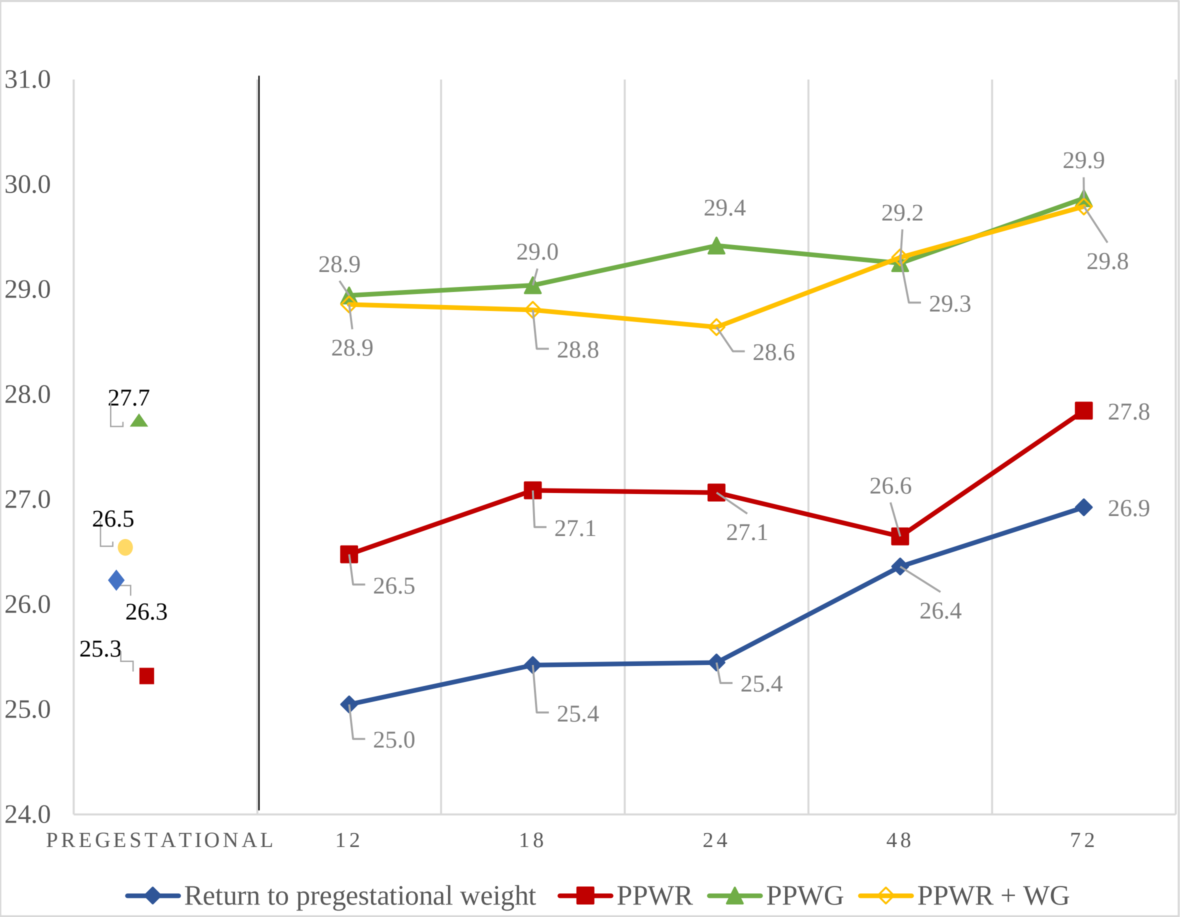

Figure S3. BMI by patterns of weight change from 12 to 72 months postpartum. The figure displays the mean BMI (kg/m^2) by patterns of weight change and postpartum month. The pregestational BMI in each group is included as a reference.

PPWG, postpartum weight gain; PPWR, postpartum weight retention; PPWR+WG, postpartum weight retention + weight gain.
